# Risk factors and management of biliary stones after living donor liver transplant and its effect on graft outcome

**DOI:** 10.3389/fmed.2022.927744

**Published:** 2022-08-23

**Authors:** Hany Dabbous, Ashraf Elsayed, Manar Salah, Iman Montasser, Mohamed Atef, Mahmoud Elmetenini

**Affiliations:** ^1^Tropical Medicine Department, Ain Shams Center for Organ Transplantation, Ain Shams University, Cairo, Egypt; ^2^Tropical Medicine Department, Ain Shams University, Cairo, Egypt; ^3^Hepatobiliary Surgical Department, Ain Shams Center for Organ Transplantation, Ain Shams University, Cairo, Egypt

**Keywords:** biliary stricture, stones, living donor liver transplant, ERCP, graft outcomes

## Abstract

**Background:**

Bile stones are associated with numerous complications in liver transplant recipients. Endoscopic retrograde cholangiopancreatography (ERCP) has been proven to be safe and highly effective in dealing with most post-transplant biliary complications.

**Objective:**

The objective of this study was to identify the possible risk factors for bile stone formation on top of biliary stricture, the effects of stones on graft outcomes, and their management.

**Methods:**

This case–control study included 83 patients who underwent living donor liver transplant (LDLT) and suffered from postoperative biliary stricture with or without stones. Patients were divided into two groups. Group 1 (*n* = 55) included patients with biliary strictures with no stones and group 2 (*n* = 28) included patients who developed stones on top of biliary strictures. Data about the recipient and donor characteristics, surgical technique, blood lipid profile, immunosuppressive drugs, post-transplant complications, and interventions were collected from the medical records.

**Results:**

The frequency of hepatitis C virus (HCV) was significantly higher in group 2 compared to group 1 (71.4% vs. 47.3%, *p* = 0.036). The body mass index (BMI) of the donors was significantly higher in group 2 than in group 1 (25.17 ± 2.53 vs. 23.68 ± 2.63, *p* = 0.015). Episodes of acute rejection were significantly higher in group 2 than in group 1 (21.4% vs. 5.5%, *p* = 0.027). The ERCP was sufficient in most of the cases (89.2%) to ensure biliary drainage. The identified independent risk factors for biliary stones included HCV, biliary drain, donor's BMI, and serum cholesterol level.

**Conclusion:**

Positive HCV, biliary drain insertion, donor's BMI, and serum cholesterol level were independent risk factors for developing bile stones on top of biliary strictures. Biliary stones were associated with high episodes of acute graft rejection, and they could be successfully managed by the ERCP modality.

## Introduction

Liver transplantation is considered the ultimate treatment for advanced chronic liver disease, acute liver failure, hepatocellular carcinoma, and inherited metabolic disorders. Although great advances have been made in the field of liver transplantation, it is still constrained by many complications (1).

Biliary complications including biliary strictures, bile stones, bile casts, bile leaks, bilomas, and hemobilia are among the common and potentially lethal problems after liver transplant, particularly living donor liver transplant (LDLT) (2).

Biliary complications in LDLT lead to a lower range of improvement in health-related quality of life compared to recipients with no biliary complications (3).

Bile stones have been recorded in 2–6% of liver transplant recipients, and they are associated with numerous complications such as recurring cholangitis, secondary biliary cirrhosis, septicemia, graft loss, and even death of the recipient (4).

The development of bile stones after liver transplantation is attributed to many risk factors but the exact pathogenesis has not been clarified yet. The development of post-transplant biliary strictures, hepatic arterial thrombosis, ischemia-reperfusion injury, and prolonged cold ischemia times have been reported as possible risk factors for bile stones (5–7). Other studies suggested an association between cyclosporine, increased blood triglycerides and cholesterol levels, and the increased tendency for bile stone formation (8, 9).

Non-invasive techniques, such as endoscopic retrograde cholangiopancreatography (ERCP) and percutaneous transhepatic cholangiography (PTC), are successful modalities that can be used for both diagnosis and treatment of bile stones (10). Instead, intervention radiology or open surgery is indicated only in a few cases where endoscopy fails (11).

No studies investigated the contributing risk factors for bile stone formation after controlling the presence of biliary strictures. Therefore, the aim of this study was to identify the possible risk factors for bile stone formation on top of biliary stricture, the effects of stones on graft outcomes, and their management.

## Materials and methods

### Study type, setting, and population

This case–control study included all patients who underwent LDLT at the Ain Shams Center of Organ Transplantation from 2009 till the end of 2020 and suffered from postoperative biliary stricture with or without stones.

All patients in this study underwent right lobe transplant with bile duct-to-duct anastomosis.

### Ethical considerations

The study was approved by the Ethics Committee of the Faculty of Medicine, Ain Shams University, Egypt (assurance no. FWA000017585).

### Inclusion criteria

We included 83 adult patients, patients aged 18 years or older, patients of both genders, and patients who underwent LDLT and suffered from postoperative biliary stricture with or without stones.

### Exclusion criteria

Patients who had hepaticojejunostomy or primary sclerosing cholangitis were excluded.

### Data collection

Patients were enrolled into two groups. Group 1 (control) included patients with biliary strictures with no stones and group 2 (cases) included patients who developed stones on top of biliary strictures.

In both groups, the following data were collected: (1) baseline characteristics of the recipients including age, sex, body mass index (BMI), the Child-Pugh score (CHILD score), the Model for End-Stage Liver Disease score (MELD score), etiology of transplantation, rectal snip plus fecal antigen for bilharziasis, and history of diabetes mellitus, hypertension, and pretransplant gall bladder stones; (2) donor characteristics including age, sex, BMI, degree of steatosis, number of biliary ducts, and biliary anastomosis; (3) surgical technique used during the transplant operation including the type of portal vein and hepatic artery anastomosis, biliary anastomosis technique, cold and warm ischemia times, and use of the biliary drain; and (4) the type of immune suppression drugs, results of lipid profile, and the recorded post-transplant complications that involved biliary leak, vascular complications (thrombosis or stenosis), cytomegalovirus infection, acute or chronic graft rejection, graft failure, and cholangitis.

As a part of the donor's evaluation, liver biopsies were evaluated by an experienced liver pathologist. Hepatic steatosis was graded quantitatively employing a 20 objective, according to the total percentage of hepatocytes involved over the 6 tissue levels. Zonal distribution of steatosis and presence of macrovesicular and microvesicular steatosis were reported.

The intraoperative biliary drains used were Nelaton drains and were usually removed after 3 months of transplantation.

Acute rejection was suspected in any recipient with unexplained elevated liver enzymes and proved by histopathology and response to increased doses of immunosuppressive drugs.

All patients were diagnosed with biliary anastomotic stricture with or without stone using imaging such as sonography and or magnetic resonance cholangiopancreatography.

All patients in both groups were managed by ERCP according to the following steps: direct cholangiography to confirm magnetic resonance cholangiopancreatography findings, evaluation of bile duct anatomy and features of the stricture (location, length, and intrahepatic anatomy), biliary sphincterotomy (if not previously performed), passage through the stricture with a 0.018-, 0.025-, or 0.035-in hydrophilic guidewire (J-shaped or straight), balloon (4, 6, or 8 mm diameter) or mechanical dilation (up to 10 Fr) of the stricture, and extraction of stones above the stricture during the index treatment using Dormia baskets or Fogarty balloons; if bile ducts cleaning was not possible, it was completed during subsequent ERCPs after dilation of the stricture, selection of stent diameter and length according to stricture morphology and anatomy of the bile ducts, and insertion of the maximum diameter of plastic stents (7, 8.5, and 10 Fr), according to stricture tightness and size of the bile ducts above and below the stricture. Finally, endoscopic re-evaluation after 3 months (stent removal, cholangiography to assess stricture morphology, extraction of sludge/casts when needed, and reinsertion of an increased number of stents if possible), removal of stents when the stricture appeared completely resolved at occluding cholangiography with a Fogarty balloon, during stent-indwelling period urgent ERCP was performed in case of cholangitis.

Full data regarding ERCP included the number of ERCP, the type and number of strictures, the presence or absence of stones and their site if present, and the number and size of stent. Furthermore, the need for PTC was recorded.

### Statistical analysis

The calculation of the sample size was based on the incidence of bile stricture in the patients who underwent LDLT, which was reported to be approximately 21.3% (12). The number of patients who underwent LDLT in our institution was 376. Therefore, the minimum sample size was 80 patients with strictures.

Data were tabulated and analyzed using the Statistical Package for Social Sciences (IBM SPSS Statistics) for Windows, version 26 (IBM Corp., Armonk, NY, USA). Categorical data were presented as numbers and percentages and the chi-square test was used to examine the relationship between two qualitative variables. Instead, Fisher's exact test was used when the expected count is < 5 in more than 20% of cells. Numerical data were tested for normality using the Shapiro–Wilk test. Parametric numerical data were presented as mean and standard deviation (SD), and the independent *t*-test was used to assess the statistical significance of the difference between the two study groups. Alternatively, non-parametric numerical data were presented as the median and interquartile range (IQR), and the Mann–Whitney test (*U* test) was applied. *P*-values < 0.05 were considered statistically significant. The univariate logistic regression analysis was carried out to identify the potential risk factors of biliary stones and clinically relevant variables that had a *p*-value < 0.1 were eligible for inclusion in the backward elimination, multivariate logistic regression analysis (13). In the multivariate analysis, the final model included independent variables that either had a Wald's test *p*-value < 0.05 or their removal affected the model significantly.

## Results

During the study period, 376 patients underwent LDLT of which 83 (22%) recipients developed biliary stricture. Patients developed either biliary stricture without stones (group 1, *n* = 55) or biliary stricture with stones (group 2, *n* = 28). The sex distribution and the means of age and BMI were comparable in both groups, with no significant differences (*p* > 0.05). Furthermore, no significant differences were detected between the study groups as regards the median CHILD and MELD scores (CHILD: 10 vs. 10, MELD: 17 vs. 16.5, respectively). Concerning the etiology of LDLT, the frequency of hepatitis C virus (HCV) was significantly higher in group 2 (71.4%) than in group 1 (47.3%, *p* = 0.036). History of diabetes mellitus, hypertension, and pretransplant gall bladder stones were homogenous in both groups (*p* > 0.05), as shown in Table 1.

**Table 1:** Baseline characteristics of the study groups.

		**Group 1 No stones**	**Group 2 stones**	**Test value**	***P*–value**
		***n* = 55**	***n* = 28**		
Age	Mean ± SD	49.13 ± 7.94	49.21 ± 9.54	−0.044^≠≠^	0.965
	Range	19–62	30–65		
Sex	Female	4 (7.3%)	3 (10.7%)	0.285^•^	0.594
	Male	51 (92.7%)	25 (89.3%)		
BMI	Mean ± SD	27.88 ± 5.13	28.43 ± 3.00	−0.525^≠≠^	0.601
	Range	17–40	22–36		
CHILD score	Median (IQR)	10 (9–11)	10 (9–11)	−0.024^≠^	0.980
	Range	5–13	8–13		
MELD score	Median (IQR)	17 (14–19)	16.5 (14.5–19)	−0.672^≠^	0.502
	Range	8–25	11–24		
Etiology	HCV	26 (47.3%)	20 (71.4%)	4.382^•^	0.036*
	HCV/HCC	20 (36.4%)	6 (21.4%)	1.924^•^	0.165
	HBV	1 (1.8%)	0 (0.0%)	0.515^•^	0.473
	AIH	2 (3.6%)	0 (0.0%)	1.043^•^	0.307
	Cryptogenic	4 (7.3%)	1 (3.6%)	0.449^•^	0.503
	Cryptogenic/HCC	2 (3.6%)	0 (0.0%)	1.043^•^	0.307
	PBC	0 (0.0%)	1 (3.6%)	1.988^•^	0.159
DM	Negative	41 (74.5%)	25 (89.3%)	2.475^•^	0.116
	Positive	14 (25.5%)	3 (10.7%)		
HTN	Negative	52 (94.5%)	26 (92.9%)	0.093^•^	0.760
	Positive	3 (5.5%)	2 (7.1%)		
Pre–transplant gall bladder stone	Negative	45 (81.8%)	21 (75.0%)	0.530^•^	0.467
	Positive	10 (18.2%)	7 (25.0%)		

SD, standard deviation; IQR: interquartile range; BMI, body mass index; CHILD, the Child–Pugh score; MELD, Model for End–Stage Liver Disease score; HCV, hepatitis C virus; HBV, hepatitis B virus; HCC, hepatocellular carcinoma; PBC, primary biliary cholangitis; AIH, autoimmune hepatitis; DM, diabetes mellitus; HTN: hypertension.

• Chi–Square test;

≠ Mann–Whitney test;

≠≠ Independent T–test.

* Significant at p < 0.05.

The technique of portal vein, as well as hepatic artery anastomosis, was not significantly different between both groups (*p* > 0.05). The biliary anastomosis technique was either right-to-left or right-to-right duct-to-duct anastomosis in both groups with no significant differences (*p* = 0.204). As well, the medians of the cold (40 vs. 45 min) and warm ischemia (45 vs. 47.5 min) times showed no significant differences between the study groups. Intraoperative biliary drain placement was 61.8% in group 1 compared with 82.1% in group 2, with no significant difference (*p* = 0.059). The prevalence of post-transplant biliary leak (20.0 vs. 7.1%), hepatic artery occlusion (12.7 vs. 10.7%), hepatic vein thrombosis (1.8 vs. 3.6%), portal vein thrombosis (3.6 each), and cytomegalovirus infection was non-significantly different between the study groups (*p* > 0.05). Episodes of acute rejection were significantly higher in group 2 (21.4%) than in group 1 (5.5%; *p* = 0.027), while the frequency of chronic rejection and graft failure was non-significantly different between the study groups (p > 0.05). The percentage of cholangitis was 47.3% in group 1 compared with 60.7% in group 2 (*p* = 0.247) (Table 2).

**Table 2:** The surgical technique used and post–transplant complications in the operation of the study groups.

		**Group 1**	**Group 2**	**Test value**	***P*–value**
		***n* = 55**	***n* = 28**		
PV Anastomosis	RPV/MPV	54 (98.2%)	28 (100.0%)	0.515^•^	0.473
	RPV/RPV	1 (1.8%)	0 (0.0%)		
HA Anastomosis	RHA+RHA	51 (92.7%)	27 (96.4%)	0.449^•^	0.503
	RHA /LHA	4 (7.3%)	1 (3.6%)		
Biliary anastomosis technique	RTD+ RTD/CBD	55(100%)	26 (92.9%)	4.593^•^	0.204
	RHD/LHD	0 (0%)	2 (7.1%)		
Cold ischemia time (minutes)	Median (IQR)	40 (30–50)	45 (35–57.5)	−1.015^≠^	0.310
	Range	15–120	20–150		
Warm ischemia time (minutes)	Median (IQR)	45 (40–60)	47.5 (35–60)	−0.151^≠^	0.880
	Range	20–120	20–80		
Biliary drain	No	21 (38.2%)	5 (17.9%)	3.563^•^	0.059
	Yes	34 (61.8%)	23 (82.1%)		
Biliary leak	No	44 (80.0%)	26 (92.9%)	2.322^•^	0.128
	Yes	11 (20.0%)	2 (7.1%)		
Vascular complication	No	45 (81.8%)	23 (82.1%)	0.300^•^	0.960
	HA occlusion	7 (12.7%)	3 (10.7%)		
	HV thrombosis	1 (1.8%)	1 (3.6%)		
	PV stenosis	2 (3.6%)	1 (3.6%)		
Cytomegalovirus infection	No	49 (89.1%)	26 (92.9%)	0.302^•^	0.583
	Yes	6 (10.9%)	2 (7.1%)		
Episode of acute rejection	No	52 (94.5%)	22 (78.6%)	4.897^•^	0.027*
	Yes	3 (5.5%)	6 (21.4%)		
Chronic rejection	No	49 (89.1%)	26 (92.9%)	0.302^•^	0.583
	Yes	6 (10.9%)	2 (7.1%)		
Graft failure	No	51 (92.7%)	28 (100.0%)	2.139^•^	0.144
	Yes	4 (7.3%)	0 (0.0%)		
Cholangitis	No	29 (52.7%)	11 (39.3%)	1.343^•^	0.247
	Yes	26 (47.3%)	17 (60.7%)		

IQR, interquartile range; PV, portal vein; HA, hepatic artery; HV, hepatic vein; RPV, right portal vein; MPV, main portal vein; RHA, right hepatic artery; LHA, left hepatic artery; RTD, right duct: CBD, common bile duct; RHD, right hepatic duct; LHD, left hepatic duct.

• Chi–Square test;

* Significant at p < 0.05.

≠ Mann–Whitney test.

Table 3 shows donor characteristics in the study groups. The mean BMI was significantly higher in group 2 (25.17 ± 2.53) than in group 1 (23.68 ± 2.63; *p* = 0.015). Otherwise, the mean age, sex distribution, the median degree of steatosis, the number of biliary ducts, and biliary anastomosis were similar in both groups (*p* > 0.05).

**Table 3:** Donor characteristics in the study groups.

**Donor data**		**Group 1**	**Group 2**	**Test value**	***P*–value**
		***n* = 55**	***n* = 28**		
Age	Mean ± SD	27.84 ± 7.05	27.96 ± 5.63	−0.083^≠≠^	0.934
	Range	18–49	18–38		
Sex	Female	8 (14.5%)	9 (32.1%)	3.528^•^	0.060
	Male	47 (85.5%)	19 (67.9%)		
BMI	Mean ± SD	23.68 ± 2.63	25.17 ± 2.53	−2.478^≠≠^	0.015*
	Range	17.7–29.6	20–29		
Degree of steatosis (%)	Median (IQR)	6 (6–8)	6 (6–8)	−0.377 ^≠^	0.706
	Range	3–15	3–15		
Number of biliary ducts	1	26 (47.3%)	8 (28.6%)	3.157^•^	0.206
	2	24 (43.6%)	15 (53.6%)		
	3	5 (9.1%)	5 (17.9%)		
Number of biliary anastomosis	1	38 (69.1%)	16 (57.1%)	0.582^•^	0.445
	2	17 (30.9%)	12 (42.9%)		

SD, standard deviation; IQR, interquartile range; No, number; BMI, body mass index.

• Chi–Square test;

≠ Mann–Whitney test;

≠≠ Independent T–test.

* Significant at p < 0.05.

There were no significant differences between the study groups regarding the medians of serum cholesterol, triglyceride, low-density lipoprotein, and high-density lipoprotein levels (*p* > 0.05). In addition, the types of the prescribed immunosuppressive drugs varied between calcineurin inhibitors, mycophenolate mofetil, and everolimus and were comparable in both groups (*p* > 0.05) (Table 4).

**Table 4:** Lipid profile and the type of immune suppression used in the study groups.

		**Group 1**	**Group 2**	**Test value**	***P*–value**
		***n* = 55**	***n* = 28**		
Serum cholesterol	Median (IQR)	112 (94–140)	126.5 (97–184.5)	−1.551^≠^	0.121
	Range	53–280	63–338		
High–density lipoprotein	Median (IQR)	34 (25–54)	35.5 (21–52.5)	−0.814^≠^	0.416
	Range	16–155	11–202		
Low–density lipoprotein	Median (IQR)	54 (43–74)	61.5 (43–77)	−0.800^≠^	0.424
	Range	12–195	5–175		
Serum triglycerides	Median (IQR)	65 (48–98)	68 (54.5–117)	−1.190^≠^	0.234
	Range	27–180	41–206		
Calcineurin inhibitors	Cyclosporin	21 (38.2%)	16 (57.1%)	2.775^•^	0.250
	Tacrolimus	32 (58.2%)	11 (39.3%)		
	Both	2 (3.6%)	1 (3.6%)		
Mycophenolate mofetil	Absent	24 (43.6%)	15 (53.6%)	0.735^•^	0.391
	Present	31 (56.4%)	13 (46.4%)		
Everolimus	Absent	39 (70.9%)	18 (64.3%)	0.378^•^	0.538
	Present	16 (29.1%)	10 (35.7%)		

IQR, interquartile range.

• Chi–Square test;

≠ Mann–Whitney test.

The median time for stone diagnosis was 6.5 months post-transplantation, while the median time for resolution was 19 months from diagnosis. Recurrence of the stones occurred in 8 patients (28.6%) with a median time of recurrence 9 months after removal. This group needed a median of 4 sessions of ERCP till resolution with 6 patients complicated by post-ERCP cholangitis, 2 patients had post-ERCP pancreatitis, and 1 patient had a migrated stent which was retrieved endoscopically (Table 5).

**Table 5:** Descriptive data regarding the stone group.

		***n* = 28**
Time of diagnosis from transplantation (months)	Median (IQR) Range	6.5 (4–13) 3–30
Time of resolution (months)	Median (IQR) Range	19 (7.5–37) 4–84
Recurrence of stones after removal	Negative Positive	20 (71.4%) 8 (28.6%)
Time of recurrence (months)	Median (IQR) Range	9 (7–35.5) 3–49
Number of ERCP sessions	Median (IQR) Range	4 (2.5–5) 2–7
Post ERCP complications	Non–complicated	19 (67.9%)
	Pancreatitis	2 (7.1%)
	Cholangitis	6 (21.4%)
	Migrated stent	1 (3.6%)

IQR, interquartile range; ERCP, Endoscopic Retrograde Cholangio–Pancreatography.

Table 6 shows the management of the study patients in both groups. A single stricture was detected in 42 (80.8%) and 23 (82.1%) patients in groups 1 and 2, respectively, while 8 (15.4%) patients in group 1 and 4 (14.3%) patients in group 2 had 2 strictures. Three strictures were detected in fewer patients (3.8 and 3.6%, respectively). Group 2 displayed a single stone (32.1%), two s*/tones (14.3%), and multiple ones in 53.6%. More than half (57.1%) had extrahepatic stones, while the stones were intrahepatic in 28.6% of patients. A stent was required in 52 (94.5%) patients in group 1 compared with 27 (96.4%) patients in group 2 (*p* = 0.705). Most patients in group 1 (61.5%) needed a single stent, while 59.3% of patients in group 2 needed 2 stents (*p* = 0.084). The most frequently used stent size was 7 in group 1 (57.7%), whereas size 10 was more frequent in group 2 (48.1%; *p* = 0.057). The median number of ERCP was significantly higher in group 2 (4) than in group 1 (2; *p* = 0.004). PTC was required in 5 (9.1%) patients in group 1 and 4 (14.3%) patients in group 2 (*p* = 0.472). Furthermore, all patients in group 1 did not require hepaticojejunostomy, while 1 (3.6%) patient in group 2 required it.

**Table 6:** Intervention in the study groups.

		**Group 1**	**Group 2**	**Test value**	***P*–value**
		***n* = 55**	***n* = 28**		
Number of ERCP	Median (IQR)	2 (2–4)	4 (2.5–5)	−2.884^≠^	0.004*
	Range	1–11	2–7		
Number of strictures	1	42 (80.8%)	23 (82.1%)	0.023^•^	0.989
	2	8 (15.4%)	4 (14.3%)		
	3	2 (3.8%)	1 (3.6%)		
Number of stones	1	–	9 (32.1%)	–	–
	2	–	4 (14.3%)		
	Multiple	–	15 (53.6%)		
Site of stones	Intrahepatic	–	8 (28.6%)	–	–
	Extrahepatic	–	16 (57.1%)		
	Both	–	4 (14.3%)		
Relation of stones to endoscopy	Before endoscopy	–	21 (75.0%)	–	–
	After endoscopy	–	7 (25.0%)		
Stent	No	3 (5.5%)	1 (3.6%)	0.143^•^	0.705
	Yes	52 (94.5%)	27 (96.4%)		
Number of stents	1	32 (61.5%)	11 (40.7%)	4.959^•^	0.084
	2	18 (34.6%)	16 (59.3%)		
	3	2 (3.8%)	0 (0.0%)		
Size of stents (fr)	7	30 (57.7%)	8 (29.6%)	5.721^•^	0.057
	10	14 (26.9%)	13 (48.1%)		
	7&10	8 (15.4%)	6 (22.2%)		
PTC	No	50 (90.9%)	24 (85.7%)	0.518^•^	0.472
	yes	5 (9.1%)	4 (14.3%)		
Hepato jejunostomy	No	55 (100.0%)	27 (96.4%)	1.988^•^	0.159
	Yes	0 (0.0%)	1 (3.6%)		
Re transplant	No	55 (100.0%)	28 (100.0%)	–	–
	Yes	0 (0.0%)	0 (0.0%)		

ERCP, Endoscopic Retrograde Cholangio–Pancreatography; PTC, percutaneous transhepatic cholangiography; IQR, interquartile range.

• Chi–Square test;

≠ Mann–Whitney test.

* Significant at p < 0.05.

Tables 7, 8 summarize the results of univariate and multivariate logistic regression analysis that was conducted to identify the risk factors for developing biliary stones following LDLT. The variables that were potential risk factors (had a *p-*value < 0.1) in the univariate analysis included positive HCV, the insertion of a biliary drain, donor's sex and BMI, and serum cholesterol level. The aforementioned factors were entered into a backward elimination, multivariate logistic regression analysis. The final model confirmed these variables to be independent risk factors except for the donor's sex that was excluded from the model.

**Table 7:** Univariate logistic regression analysis for determining potential risk factors of developing biliary stones post–LDLT (total *n* = 83).

**Variables**	***P*–value**	**OR**	**95% CI for OR**
Age (years)	0.964	1.001	0.948–1.057
Sex (Male vs. Female)	0.596	0.654	0.136–3.147
BMI (Kg/m2)	0.597	1.028	0.929–1.137
CHILD score	0.615	1.071	0.820–1.399
MELD score	0.489	1.043	0.926–1.174
HCV	0.039	2.788	1.051–7.400
HCV/HCC	0.170	0.477	0.166–1.373
Diabetes Mellitus	0.127	0.351	0.092–1.345
Hypertension	0.761	1.333	0.210–8.481
Pre–transplant gall bladder stone	0.468	1.500	0.501–4.488
Hepatic artery anastomosis	0.512	0.472	0.050–4.438
Cold ischemia time (min)	0.400	1.008	0.989–1.028
Warm ischemia time (min)	0.676	0.994	0.964–1.024
Biliary drain (Yes vs. No)	0.065	2.841	0.937–8.618
Biliary leak (Yes vs. No)	0.144	0.308	0.063–1.498
Vascular complications (Yes vs. No)	0.971	0.978	0.299–3.200
Cytomegalovirus infection (Yes vs. No)	0.585	0.628	0.118–3.335
Acute rejection (Yes vs. No)	0.039	4.727	1.084–20.618
Chronic rejection (Yes vs. No)	0.585	0.628	0.118–3.335
Graft failure (Yes vs. No)	0.999	0.000	0.000 –
Cholangitis (Yes vs. No)	0.249	1.724	0.684–4.347
Donor's age (years)	0.933	1.003	0.936–1.075
Donor's sex (Male vs. Female)	0.066	0.359	0.121–1.070
Donor's BMI (Kg/m^2^)	0.019	1.251	1.038–1.509
Degree of steatosis	0.605	0.958	0.814–1.128
No. of biliary ducts			
2 ducts vs. 1 duct	0.065	2.683	0.941–7.652
3 ducts vs. 1 duct	0.076	3.857	0.867–17.158
No.of anastmosis (2 vs. 1)	0.282	1.676	0.654–4.300
Serum cholesterol	0.038	1.010	1.001–1.019
High density lipoprotein	0.485	1.005	0.992–1.018
Low density lipoprotein	0.625	1.003	0.990–1.017
Serum triglycerides	0.125	1.009	0.998–1.020
Calcineurin inhibitors			
Cyclosporin vs. both	0.740	1.524	0.127–18.324
Tacrolimus vs. both	0.769	0.688	0.057–8.344
Mycophenolate mofetil (present vs. absent)	0.392	0.671	0.269–1.674
Everolimus (present vs. absent)	0.539	1.354	0.515–3.563

BMI, body mass index; CI, confidence interval; HCC, hepatocellular cell carcinoma; HCV, hepatitis C virus; OR, odds ratio; significant at p < 0.05.

**Table 8:** Backward elimination, multivariate logistic regression analysis for determining independent risk factors of developing biliary stones post–LDLT (total *n* = 83).

**Models**	**Variables**	***P*–value**	**OR**	**95% CI for OR**
Model 1	HCV	0.065	2.878	0.936–8.843
	Biliary drain (Yes vs. No)	0.039	4.007	1.075–14.927
	Donor's sex (Male vs. Female)	0.252	0.468	0.128–1.714
	Donor's BMI (Kg/m^2^)	0.035	1.239	1.015–1.513
	Serum cholesterol	0.025	1.012	1.001–1.022
Model 2	HCV	0.038	3.189	1.066–9.540
	Biliary drain (Yes vs. No)	0.044	3.727	1.033–13.442
	Donor's BMI (Kg/m^2^)	0.037	1.239	1.012–1.516
	Serum cholesterol	0.013	1.013	1.003–1.023

CI, confidence interval; HCV, hepatitis C virus; OR, odds ratio; significant at p < 0.05.

## Discussion

Biliary complications substantially influence the morbidity and mortality outcomes of liver transplantation with an associated mortality of 10% (14). The overall incidence of biliary complications ranges between 15 and 25% (2). This incidence has declined in deceased-donor liver transplants but continued to be high in LDLT (15).

Liver transplantation may be complicated by hypoperfusion and ischemic injury of the biliary tract that manifest as either anastomotic or non-anastomotic strictures, or both. The presence of strictures predisposes to the development of stones. Then, the formation of stones may result in biliary obstruction, jaundice, cholangitis, and liver cell injury (16).

Therefore, the aim of this study was to identify the possible risk factors for bile stone formation on top of biliary strictures, the effects of stones on graft outcomes, and their management.

During the study period, 83 (out of 376 patients who underwent LDLT) patients developed biliary stricture, representing 22% of all recipients. This finding agrees with Sarhan et al. (7) who reported an overall stricture rate of 21.7% in patients with LDLT. It is also consistent with Kim (17) in which biliary stricture occurred in 19.5% of recipients. Previous studies have reported ranges between 8.3 and 31.7% (18, 19).

The formation of biliary stones and sludge can occur at any time after liver transplantation (11). In this study, the median time for stone formation was 6.5 months after liver transplant in contrast to Moy and Birk (2) who found it at 19 months and Eminler et al. (20) who found it at 26 months.

Relatively, early detection of biliary stones may be related to multiple risk factors in the same patient and the successful follow-up program that allowed us to detect the problem early.

The formation of biliary stones is mainly multifactorial. Many inflammatory, physical, and metabolic factors might predispose to bile stone formation by increasing the bile viscosity or decreasing the bile flow (4). Earlier studies reported that the coexistence of biliary stricture and high cholesterol triglyceride levels is independent risk factors for bile stone formation (9, 21). Furthermore, Kirnap et al. (4) found an increased incidence of bile stones in patients who had biliary stricture or leakage, recurrent cholangitis, high blood cholesterol and triglyceride levels, and prolonged cold ischemia time. In addition, a link has been reported between post-transplant biliary stone formation and immune suppression by cyclosporine as well as biliary mucosal damage due to ischemia or infection (22).

Previous studies have also shown that inadequate surgical technique, multiple biliary reconstructions, old donor age, cytomegalovirus infection, chronic rejection, and early HCV recurrence are associated with biliary complications (6, 18, 23).

Compared to previous research, this study detected significant associations between the formation of biliary stones and HCV infection, the insertion of a bile drain, and the high BMI of the donors. Alternatively, we did not find associations between the recipients' age, sex, BMI, the severity of liver disease (CHILD and MELD scores), the presence of chronic medical conditions such as diabetes and hypertension or having pretransplant gall bladder stones, and the formation of post-transplant biliary stones. Furthermore, the surgical techniques including portal vein, hepatic artery, or biliary anastomosis did not show significant relationships with stone formation. Similarly, warm and cold ischemia times, as well as blood lipid profile (e.g., triglyceride, low-density lipoprotein, and high-density lipoprotein levels), did not show any significant impact on stone formation.

Cytomegalovirus infection and its relationship with a higher occurrence of biliary complications have been identified by Gotthardt et al. (24). In contrast, our study showed that 6 (10.9%) patients in group 1 and 2 (7.1%) patients in group 2 had cytomegalovirus infection with no significant difference between the two groups.

Regarding immunosuppression post-LDLT, cyclosporine inhibits bile acid synthesis and, therefore, increases the probability of stone formation (2). However, in our study, cyclosporine was used more in group 2 than in group 1 (57.1% vs. 38.2) but with no significant statistical difference.

In this study, intraoperative biliary drain placement was more in group 2 than in group 1 (82.1 vs. 61.8%) than in group 2, with no significant statistical difference in univariate analysis, but with a significant increase in the risk on multivariate analysis. At present, there is sufficient evidence that the use of T-tube drainage in biliary reconstruction does not significantly alter the risk of biliary stricture after liver transplantation. Moreover, the presence of this drainage may increase the risk of infection, leak, and metabolic complications (25).

Although ischemia due to vascular complications was a well-known risk factor for stone formation according to Lee et al. (11), in this study, there was no significant difference between the two groups regarding hepatic artery and portal vein occlusion.

Participants in the control group were patients without stones but had strictures. In contrast, in the abovementioned previous studies, controls without stones were chosen randomly from all patients who underwent liver transplantation. The observed differences between the control groups might explain the absence of significant differences between patients with and without stone formation.

Furthermore, it seems that HCV infection and high BMI of the donors (median BMI was 25.17 kg/m^2^ in group 2 and 23.68 kg/m^2^ in group 1) were the only significant risk factors for developing bile stones on top of biliary strictures. A systematic review that analyzed 11 observational studies showed that donor BMI in LDLT had no influence on long-term outcomes including graft and patient survival, but high donor BMI was associated with a higher rate of macrosteatosis. Another study investigated the risk factors for bile stones in grafts with biliary strictures and concluded that the female sex of the recipient was the main risk factor (26).

The aim of this study was also to identify the effects of biliary stones on graft outcomes. Episodes of acute rejection were significantly more frequent in group 2 than in group 1 (21.4 vs. 5.5%), but there were no significant differences regarding chronic rejection or graft failure. Some studies showed that graft survival was not influenced by the onset of bile stone formation (26) or the development of anastomotic biliary complications (26). However, Rönning et al. (27) reported a significantly impaired graft survival in patients with biliary complications compared with patients without biliary complications.

Biliary stones after LDLT can be managed by either therapeutic ERCP, PTC, or surgery; however, endoscopic therapy is the preferred approach for disease management in the majority of transplantation departments, with the PTC primarily reserved for cases of ERCP failures (28).

All recipients in our study underwent ERCP through papilla (regular ERCP), and they all had choledochocholedochostomy. ERCP was sufficient in most cases to ensure biliary drainage in 74 (89.2%) recipients, and only 9 (10.8%) recipients needed percutaneous biliary drainage. This coincides with Na et al. (28) who reported a successful stent insertion through the ERCP in 81.5% of cases, while 12 (18.5%) patients underwent percutaneous biliary drainage. Endoscopic treatment was difficult in these patients because of the tight anastomotic stricture. Eminler et al. (20) also reported that in 13 out of 16 (81.2%) patients with LDLT, ERCP was adequate, and the anastomosis was traversed by a guidewire inserted through the papilla, but a percutaneous route was required in the remaining 3 patients. In patients with distal stones, Spier et al. (9) showed that a single ERCP session with biliary sphincterotomy followed by balloon or basket extraction was sufficient in 59–66% of cases. Alternatively, Kirnap et al. (4) reported that 21% of patients with bile stones had ERCP, while others had repeated percutaneous transhepatic procedures.

Biliary stones are usually extracted by using a balloon and/or basket and most patients with an anastomotic stricture require ongoing ERCP sessions every 3–4 months with balloon dilation and long-term stenting with an increasing number of stents and stent diameter (29).

Regarding the number of ERCP sessions, recipients with biliary stones on top of biliary stricture needed a significantly higher number of ERCP sessions than recipients with biliary stricture alone (median of 4 sessions vs. 2 sessions). This is consistent with Eminler et al.'s (20) findings, in which LDLT patients with biliary stones on top of anastomosis stricture had a median of 3 ERCP sessions.

Studies comparing ERCP with dilation alone with ERCP with dilation and stent placement have concluded that serial dilation with stent placement leads to higher success rates (41 vs. 75%) (29).

In this study, post-ERCP cholangitis occurred in 6 (21.4%) patients of the stone group, while Eminler et al. (20) reported 2 incidences of post-ERCP pancreatitis (12.5%) in the LDLT group.

This study is limited by being a single-center study that included a limited number of patients, which is probably due to including only patients with strictures with and without stone formation, although it is the first study that explored the contributing risk factors for biliary stone formation on top of strictures in comparison with controls with strictures only in patients with LDLT.

We need more collaboration with other liver transplantation centers to perform a multicenter study in order to obtain a more clear picture about the possible risk factors of stone formation and methods of management of such a complication.

In conclusion, positive HCV, biliary drain insertion, donor's BMI, and serum cholesterol level were independent risk factors for developing bile stones on top of biliary strictures. Biliary stones were associated with high episodes of acute graft rejection, but they had no significant impact on chronic rejection or graft failure. In addition, biliary stones could be successfully managed by the ERCP modality in most cases (89.2%). However, the total number of ERCP sessions was much higher in recipients with biliary stones on top of strictures than those with biliary stricture alone.

## Data availability statement

The original contributions presented in the study are included in the article/supplementary material, further inquiries can be directed to the corresponding author.

## Ethics statement

The studies involving human participants were reviewed and approved by the Ethical Committee of Ain Shams University Hospitals (Cairo, Egypt) in accordance with the Local Research Governance Requirements. Written informed consent for participation was not required for this study in accordance with the national legislation and the institutional requirements.

## Author contributions

HD treated the patient as endoscopists. MS, HD, and MA reviewed the literature and contributed to manuscript drafting. ME was the surgeon responsible for the transplanation operation. AE was responsible for revision of the manuscript for important intellectual content. IM was responsible for revising the manuscript drafting. All authors issued final approval of the version to be submitted. All authors have given final approval of the version of the article to be published.

## Conflict of interest

The authors declare that the research was conducted in the absence of any commercial or financial relationships that could be construed as a potential conflict of interest.

## Publisher's note

All claims expressed in this article are solely those of the authors and do not necessarily represent those of their affiliated organizations, or those of the publisher, the editors and the reviewers. Any product that may be evaluated in this article, or claim that may be made by its manufacturer, is not guaranteed or endorsed by the publisher.

## Abbreviations

ERCP: Endoscopic Retrograde Cholangiopancreatography
LDLT: living donor liver transplant
MELD: Model for End-Stage Liver Disease
PTC: Percutaneous Transhepatic Cholangiography.
